# A Laboratory Protocol for Routine Therapeutic Drug Monitoring of Beta-Lactams Antimicrobials in Horses and Dogs

**DOI:** 10.3390/antibiotics14040390

**Published:** 2025-04-09

**Authors:** Anisa Bardhi, Aliai Lanci, Aurora Mannini, Carolina Castagnetti, Andrea Barbarossa

**Affiliations:** 1 Department of Veterinary Medical Sciences, University of Bologna, 40064 Ozzano dell'Emilia, Bologna, Italy; anisa.bardhi@unibo.it (A.B.); aliai.lanci2@unibo.it (A.L.); aurora.mannini@unibo.it (A.M.); carolina.castagnetti@unibo.it (C.C.); 2 Health Sciences and Technologies-Interdepartmental Centre for Industrial Research (CIRI-SDV), University of Bologna, 40064 Ozzano dell’Emilia, Bologna, Italy

**Keywords:** therapeutic dosage, beta-lactams, ampicillin, sulbactam, animals, liquid chromatography, tandem mass spectrometry, LC-MS/MS

## Abstract

**Background:** Although antibiotic resistance is a well-known issue in veterinary medicine, studies proposing real-time therapeutic monitoring (TDM) are lacking. The objective of the present study was to develop a simple and rapid protocol for the real-time therapeutic monitoring of antibiotics in horses and dogs. **Methods:** A reliable TDM protocol should encompass guidelines for the definition of plasma/serum collection time points, sample management by the clinical staff, transportation to the laboratory, and the availability of robust and swift analytical technologies. Ampicillin and sulbactam were quantified using liquid chromatography–tandem mass spectrometry (LC-MS/MS) in the plasma or serum of animals treated with ampicillin alone or combined with sulbactam. **Results:** The method was successfully applied to samples collected from animals hospitalized in our veterinary hospital and proved helpful in understanding the pharmacokinetics of this antibiotic in critically ill patients. **Conclusions:** Combined with minimum inhibitory concentration (MIC) data, this approach enables PK/PD evaluations to support the development of personalized therapeutic strategies and optimized dosing regimens for animals.

## 1. Introduction

Effectively addressing infections in intensive care units represents a difficult task [1,2], especially in veterinary medicine. This challenge is exacerbated by the persistent issue of antimicrobial resistance, a major concern impacting both human and animal health globally [3,4]. With an increasing emphasis on promoting responsible antibiotic usage and addressing its implications [5], the therapeutic drug monitoring (TDM) of antibiotics has become a standard practice in most human medical facilities [6,7,8,9,10]. This evolution serves as a valuable tool for healthcare practitioners in ensuring responsible and effective antibiotic management. The selection of appropriate antibiotic therapy and the correct dosage represent the two fundamental elements of TDM [11,12,13,14]. Typically, the optimization of treatment, including the choice of antibiotic class, administration route, and dosage, is guided by monitoring the selected antibiotics in patients’ plasma or serum [8,13]. Indeed, one of the primary prerequisites for conducting a TDM study is the use of highly efficient and robust analytical methods. The approaches for the accurate quantification of target antibiotics in animal biological fluids must be validated in accordance with the guidelines set by the European Medicines Agency (EMA) [15] or the Food and Drug Administration (FDA) [16]. Furthermore, micro-sampling techniques, such as dried blood spots (DBSs), are increasingly recommended as alternative sampling methods for TDM due to advantages such as non-invasive sample collection and the need for smaller blood volumes [17,18,19].

In the contemporary landscape, liquid chromatography coupled with tandem mass spectrometry (LC-MS/MS) stands out as an accurate and sensitive technique for antibiotic quantification and a continually expanding range of clinical applications [20,21,22,23]. These include TDM, where efficient methods that facilitate rapid turnaround times are crucial to help the antimicrobial stewardship team quickly adopt and optimize treatment strategies [6,24].

In recent years, several studies quantifying different antibiotics in healthy and hospitalized animals for pharmacokinetic (PK) and pharmacokinetic/pharmacodynamic (PK/PD) purposes have been published [25,26,27,28,29,30]. However, laboratory protocols for routine TDM of antibiotics are poorly documented in companion animals, despite the common use of certain classes in critical care settings [31,32,33,34,35]. In particular, there is limited data on optimizing ampicillin dosage in dogs and horses [25,26], and currently, no information is available on ampicillin dosing and pharmacokinetics in critically ill foals.

To our knowledge, antibiotic TDM assays for use in routine clinical practice with companion animals have not yet been validated. This study aimed to establish a clinical and laboratory protocol for the TDM of two widely used beta-lactams—namely, ampicillin and piperacillin (with or without beta-lactamase inhibitors such as sulbactam and tazobactam)—in dogs and horses. This involved identifying the most strategic sample collection time points and developing an LC-MS/MS method capable of quantifying these drugs in blood-derived matrices such as plasma, serum, and DBSs.

## 2. Results

### 2.1. Method Validation

**Figure 1:**
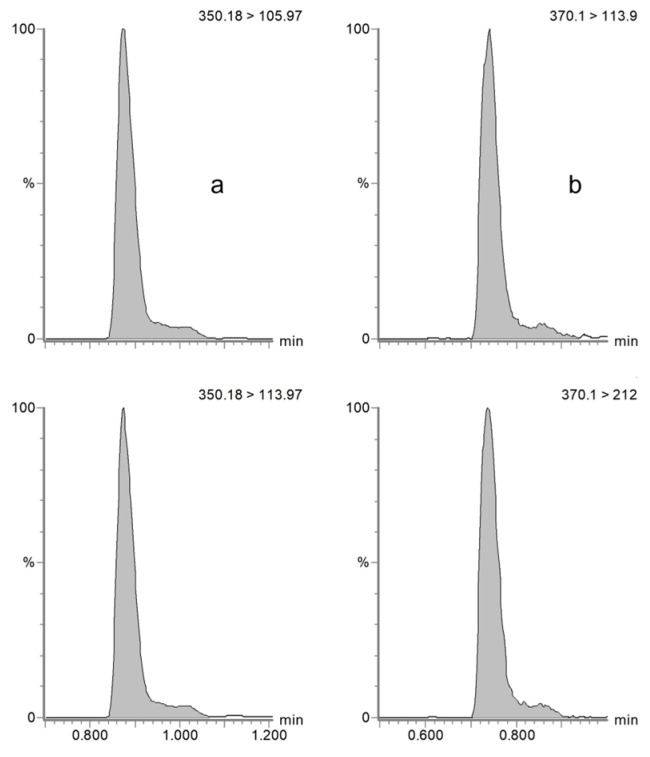
Chromatograms obtained from the MRM analysis of ampicillin (**a**) and the internal standard amoxicillin-d4 (**b**) in a horse plasma sample.

**Figure 2:**
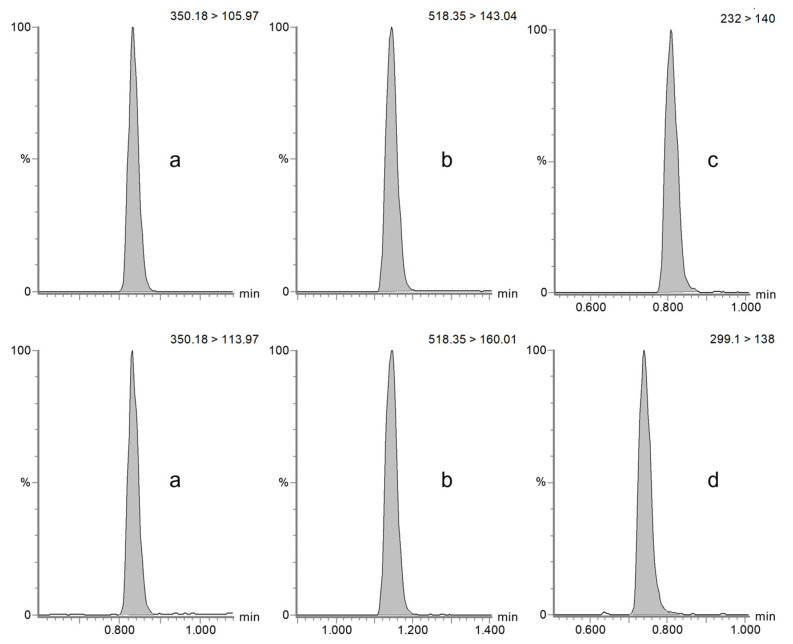
Chromatograms obtained from the MRM analysis of ampicillin (**a**), piperacillin (**b**), sulbactam (**c**), and tazobactam (**d**) in dog serum samples.

The specificity and selectivity of the method were assessed by confirming the absence of interfering peaks at the retention time of the target compounds and the internal standard amoxicillin-d4 in horse plasma (see Figure 1) and dog serum (see Figure 2) and by injecting six blank samples of each matrix of interest.

Calibration curves in both horse plasma and dog serum, prepared over three separate testing days, consistently exhibited a coefficient of determination (r^2^) ≥ 0.99 for both species. Furthermore, calibrators consistently fell within ±15% of the expected value, demonstrating the linearity of the method across the tested concentration intervals. Specifically, the calibration range 0.1–100 µg/mL was validated for ampicillin in horse plasma and dog serum. The calibration range for sulbactam in dog serum was 0.5–100 µg/mL. Accuracy and precision at all QC levels, in both intraday and interday conditions, are shown in Table 1 (ampicillin in horses and dogs) and Table 2 (sulbactam in dogs).

The results of ampicillin and (only for dog) sulbactam ME, RE, and PE experiments (described in Section 4.5.4) are reported in Table 3.

The repeated injection of blank samples following the ULOQ did not produce any detectable chromatographic signals.

The results of the experiment conducted to assess the short- and long-term stability of ampicillin in equine plasma are reported in Table 4.

### 2.2. Method Application

The method was successfully applied to plasma samples collected from four horses and 10 foals and serum samples collected from two dogs hospitalized at our veterinary hospital.

The aim was to monitor the therapeutic effect of ampicillin in these ill subjects. Plasma samples were collected primarily 5 min before and 5 min after the administration to assess the through and peak ampicillin concentrations. The residual concentrations of ampicillin were in the 0.473–1.621 µg/mL range (median 1.117 µg/mL) in horses and in the 0.245–5.870 range (median 1.453 µg/mL) in foals. Maximal concentrations were between 64.875 and 85.081 µg/mL (median 73.386 µg/mL) in horses and between 16.175 and 75.710 µg/mL (median 55.129 µg/mL) in foals.

## 3. Discussion

The growing threat of antimicrobial resistance underscores the need for precise dosing of antimicrobial drugs to effectively combat resistant pathogens [4,36]. Overprescription, often driven by diagnostic uncertainties or insufficient knowledge of optimal dosages, has exacerbated this issue. Furthermore, suboptimal dosing may prevent the attainment of the minimum inhibitory concentrations (MICs) necessary for effective treatment, compromising therapeutic outcomes [37,38,39]. Optimizing treatment involves selecting the most appropriate antibiotic, ensuring adequate drug exposure in relation to microbial susceptibility, and minimizing adverse effects to improve patient adherence to the prescribed regimen [6].

In veterinary medicine, the establishment of rational target plasma antibiotic concentrations is still lacking. Therapeutic ranges for animals are often extrapolated from human medicine [40], highlighting the need for reliable and accurate methods for antimicrobial monitoring. Current techniques, such as immunoassays and traditional high-performance liquid chromatography (HPLC) methods [41,42,43,44], are hindered by labor-intensive sample preparation and limited sensitivity and specificity [45,46]. These analytical challenges have been overcome with the introduction of liquid chromatography–tandem mass spectrometry [47,48,49]. Indeed, LC-MS/MS is increasingly being integrated into veterinary practice, both for research purposes [27,28,29,50,51] and routine clinical investigations [52].

However, to date, a standardized laboratory protocol for TDM in veterinary practice is lacking, and real-time TDM services are typically unavailable in veterinary hospitals. Although studies have evaluated the use of ampicillin in animals [25,26], none have specifically addressed a protocol for ampicillin TDM in companion animals. This gap highlights the need for validated methodologies in veterinary medicine to ensure optimal dosing and improve clinical outcomes.

In response to this, we developed an LC-MS/MS method for the quantification of some of the most commonly used antibiotics in dogs and horses. Initially, the approach was optimized for the determination of ampicillin, sulbactam, piperacillin, and tazobactam in plasma and serum. However, given the restriction of piperacillin and tazobactam combinations in veterinary medicine [53], we decided to focus here on the validation data for ampicillin.

During method development, we also explored the use of the DBS micro-sampling technique. This offers several advantages, including low blood volume requirements, cost-effectiveness, and ease of transport [54,55]. It may be especially beneficial for small animals weighing less than 5 kg, where repeated blood sampling is challenging [56,57]. Since plasma and serum remain the primary matrices for establishing routine TDM applications, we decided to first focus on the traditional liquid matrices. However, the preliminary laboratory tests conducted for the four antibiotics in DBSs yielded promising results. Further validation and in vivo studies are planned to assess the practicality of DBSs in clinical settings.

Once the analytical method was validated, we explored the feasibility of integrating the LC-MS/MS method into routine veterinary care by establishing a TDM service in our laboratory to assist the clinical staff in making informed therapeutic decisions based on real-time antibiotic levels. The first step was to establish a simple sampling protocol for routine TDM once steady-state drug levels had been achieved, typically after 4–5 half-lives [7]. Given the time-dependent nature of β-lactams antibiotics, we collected two samples: one to evaluate the trough concentration (taken 5 min before the next dose) and one for peak concentration (taken 5 min after administration). This sampling schedule is easily manageable for clinical staff, providing valuable insights into residual and peak antibiotic concentrations and helping determine if drug levels fall within the therapeutic range. Ampicillin was chosen due to its broad therapeutic range, low incidence of toxicity, and availability for intravenous administration. In equine medicine, ampicillin is considered a drug of choice for treating infections in both neonatal foals and adult horses [57,58]. Accordingly, this antimicrobial is classified as a first-line antimicrobial (category D) by the European Medicines Agency’s Antimicrobial Advice Ad Hoc Expert Group (AMEG), making it an important tool in treating conditions with low resistance risk [58]. This is particularly relevant for infections by *Streptococcus equi* subsp. *zooepidemicus* and suspected septic conditions [59], as supported by a 2016 study, which demonstrated that 91.5% of bacteria isolated from septic foals were susceptible to the ampicillin–amikacin combination [60].

This investigation was also valuable for calibrating our analytical method to detect both low and high concentrations. The UHPLC-MS/MS method described here was validated following the EMA [15] and FDA [16] guidelines, with a range of 0.1–100 µg/mL (LLOQ-ULOQ) for ampicillin in horses and dogs and 0.5–100 µg/mL (LLOQ-ULOQ) for sulbactam in dogs. These ranges were found to be suitable for the analysis of plasma samples collected from hospitalized horses and newborn foals. Thanks to the high analytical sensitivity, ampicillin and sulbactam were consistently quantifiable, providing effective support in critical care settings. The validated UHPLC-MS/MS method proved to be simple, accurate, and suitable for TDM applications in different species.

Considering that sample volume is often a limitation when working with small-sized animals, such as dogs and cats, we validated our analytical approach using 100 µL of plasma or serum. The sample preparation procedure, consisting of protein precipitation followed by centrifugation and dilution in a vial, makes the technique very simple and quick to perform. Additionally, the short analytical run (only 4 min) ensures fast analysis and availability of results in a short time. The overall features of the analytical method may enable laboratory staff to perform these analyses twice daily for routine TDM purposes.

Furthermore, based on the short-time stability tests conducted in our laboratory, plasma samples fortified with ampicillin were found to be stable for 1, 3, 6, and 24 h when stored at 4 °C. These findings are crucial for managing routine TDM using LC-MS/MS, as they minimize costs and enable equipped laboratories to extend this service to external hospitals or practitioners lacking access to advanced analytical facilities. Additionally, the long-term stability results for plasma samples stored for 40 days at −20 °C and −80 °C suggest that retrospective studies and long-distance collaborations are feasible.

To our knowledge, the present study is the first to introduce a TDM protocol that begins with sample handling and extends to laboratory management for ampicillin quantification (also applicable to other beta-lactams antibiotics) in veterinary medicine. However, the small sample size of the patients enrolled in this preliminary application is not representative of the diversity within these species. This represents a limitation, primarily due to the challenges associated with enrolling critically ill hospitalized patients and collecting their samples, although the primary focus of this study was to introduce a protocol for the therapeutic drug monitoring of antibiotics in animals. In future studies, more strictly focused on the therapeutic drug monitoring for specific drugs and clinical scenarios, we plan to increase the number of both patients enrolled and samples collected to ensure statistical robustness and allow deeper evaluations.

In perspective, collaboration between different laboratory units is essential for obtaining comprehensive results and facilitating therapeutic evaluations in TDM studies and routine practice. Integrating data on antibiotic plasma concentrations with other biochemical and hematological parameters, along with MIC values of pathogens, would enable a more accurate understanding of the patient’s condition. In this context, once sufficient PK data are collected from a relevant number of subjects and covariates such as serum albumin and creatinine are considered, our goal is to develop PK models. Combining these data with MIC values will enable PK/PD simulations, offering deeper insights into therapeutic effects and helping in the optimization of dosing regimens for patients.

## 4. Materials and Methods

### 4.1. Materials

**Figure 3:**
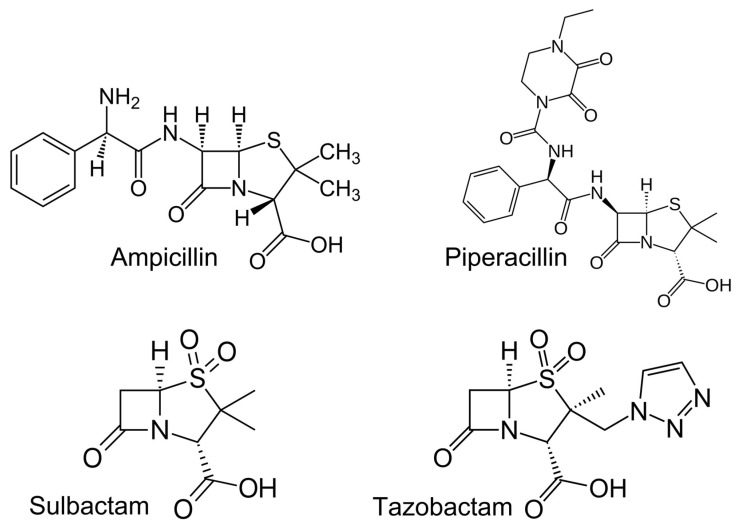
Chemical structure of ampicillin, piperacillin, sulbactam, and tazobactam.

Analytically pure standards of ampicillin, piperacillin, sulbactam, tazobactam, and amoxicillin-d4 (used as internal standard, IS) were purchased from Toronto Research Chemicals (Toronto, ON, Canada). The chemical structures of target analytes are reported in Figure 3.

Acetonitrile (ACN), formic acid (FA), and methanol (MeOH), all of LC-MS/MS grade, were obtained from Merck (Milan, Italy). Ultra-pure water (H_2_O) was freshly produced in-house using the Sartorius, Arium^®^ Ultrapure Water Systems (Varedo, Italy).

Whatman 903 Protein Saver Cards were used for spotting the blood samples and purchased from Merck (Milan, Italy).

Drug-free blood collected from healthy animals from the blood bank of our veterinary hospital was used for method development and validation.

### 4.2. Stock and Working Solutions

Individual stock solutions of all antibiotics were freshly prepared each day of analysis at a concentration of 1000 µg/mL dissolving 5 mg of each powder in 5 mL of H_2_O. Working solutions at 1, 5, 20, 50, 200, 500, and 1000 µg/mL, to be used for calibrators and quality control samples, were obtained by serial dilution of the stock solutions with H_2_O.

A stock solution of amoxicillin-d4 at 100 µg/mL (used as an internal standard, IS) was obtained by dissolving 5 mg of pure powder in 50 mL of ACN:H_2_O 85:15 (*v*/*v*) and was stored at −20 °C.

### 4.3. Sample Preparation

#### 4.3.1. Plasma/Serum

Plasma/serum samples were prepared following the procedure previously reported [29]. Briefly, 100 µL of matrix was added to 200 µL of acetonitrile (ACN) and 10 µL of IS (5 µg/mL in ACN). Samples were then vortex-mixed for 30 s and centrifuged for 10 min at 21,000× *g* at 20 °C. Finally, 10 µL of supernatant were transferred in an LC vial containing 990 µL of 0.1% FA aqueous solution and injected.

#### 4.3.2. Dried Blood Spot

For the extraction of DBS samples, the whole spot (20 μL) was cut out of the Whatman 903 Protein Saver Card with scissors and transferred to a 1.5 mL Eppendorf microtube containing 400 μL of a 30:70 solution of H_2_O:ACN (*v*/*v*) and 10 μL of IS. The microtubes were vortex-mixed for 1 min and then placed in an ultrasonic bath for 30 min. After centrifugation (10 min, 21,000× *g*, 20 °C), an aliquot of 100 μL was transferred into an LC vial containing 200 μL of 0.1% FA in H_2_O.

### 4.4. Liquid Chromatography–Tandem Mass Spectrometry (LC-MS/MS)

Ultra-high performance liquid chromatography (UHPLC) was carried out on a Waters Acquity UPLC^®^ system (Milford, MA, USA) configured with a binary pump, thermostated autosampler, and column oven. Chromatographic separation was achieved using a Waters Acquity BEH C18 (50 × 2.1 mm, 1.7 μm) column protected by the corresponding VanGuard pre-column (Waters, Milford, MA, USA), held at a temperature of 35 °C. All target analytes were separated using a 4 min chromatographic run under programmed conditions, utilizing 0.1% FA in H_2_O (solvent A) and ACN (solvent B) at a flow rate of 0.4 mL/min. Extracted samples were kept in the autosampler at 20 °C, and 5 μL from each vial was injected in the analytical system. The UHPLC was coupled to a Waters XEVO TQ-S Micro triple quadrupole mass spectrometer (Waters, Milford, MA, USA), using positive electrospray ionization (ESI+) for ampicillin, amoxicillin-d4, and piperacillin and ESI- for sulbactam and tazobactam. The instrument operated in multiple reaction monitoring (MRM) mode, monitoring the most abundant fragment ion for each analyte. For ampicillin, piperacillin, and amoxicillin-d4, a second transition was monitored for confirmation (data are reported in Table 5).

Data acquisition and processing were performed using the instrument’s proprietary software, MassLynx 4.2 (Waters, Milford, MA, USA).

### 4.5. Method Validation

The method was validated following the guidelines set by the EMA and FDA [15,16] and tested on three separate days. The considered parameters were specificity and selectivity, matrix effect, recovery, process efficiency, linearity, accuracy and precision, calibration range, stability, and carry-over.

#### 4.5.1. Specificity and Selectivity

Chromatographic conditions were optimized by injecting individual pure solutions at 0.01 µg/mL of each analyte, defining their specific retention times. The selectivity of the method was then assessed by analyzing 6 blank samples of equine plasma, canine serum, and canine DBSs, verifying the absence of chromatographic signals around the elution time of each analyte.

#### 4.5.2. Calibration Range

Matrix-matched calibration curves were freshly prepared for each day of validation. These curves included a blank sample, a zero point (spiked with IS), and calibrators at 7 concentrations (0.1, 0.5, 2, 5, 20, 50, and 100 µg/mL). Peak area ratios between target analytes and amoxicillin-d4 (IS) were plotted against their concentration, and a linear least square regression model was applied. All calibration standards should be within ±15% of the nominal concentration, and the resulting coefficient of correlation (r^2^) was considered acceptable if ≥0.99.

The lower limit of quantification LLOQ was defined as the lowest concentration measured in the samples that could be detected with a signal-to-noise (S/N) ratio ≥ 10 and acceptable accuracy (within ±20%) and precision (CV < 20%) after the injection of four replicates.

#### 4.5.3. Accuracy and Precision

The intra- and interday accuracy and precision of the method for ampicillin and sulbactam were evaluated in both species by preparing quality control (QC) samples in 5 replicates at 0.1 µg/mL (LLOQ), 0.5 µg/mL (QCL), 5 µg/mL (QCM), and 50 µg/mL (QCH).

Accuracy, expressed as relative difference between measured value and expected concentration, was considered acceptable if within ±15% of the nominal concentration. Similarly, precision, defined as the coefficient of variation (CV%) among repeated individual measures, had to be <15%.

#### 4.5.4. Matrix Effect, Recovery, Process Efficiency

Matrix effect (ME), extraction recovery (RE), and process efficiency (PE) were evaluated following the method described by Matuszewski et al. [61]. In this approach, peak areas obtained from three types of samples, each prepared in 3 replicates, are compared: (A) standard calibrators in mobile phase, containing the same amount of target analytes as QC samples; (B) blank samples extracted as described above and added with the same amount of each analyte (post-extraction); (C) samples fortified with the same amount of each analyte and then extracted (pre-extraction) as described above.

Drug-free matrices collected from 6 different subjects were used to prepare these samples, allowing us to also assess the potential subject-related differences. Then, ME, RE, and overall PE were calculated through comparison of the analytical response obtained from the three types of samples described above, according to the following formulas:

$$ME=\frac{B}{A}\;(\%)$$

$$RE=\frac{C}{B}\;(\%)$$

$$PE=\frac{C}{A}\;\left(\%\right)$$

#### 4.5.5. Carry-Over

The absence of carry-over contamination was assessed by analyzing 6 drug-free plasma and serum samples after the injection of the ULOQ. The analytical response in the blank samples had to be below 20% of the LLOQ for all target compounds.

#### 4.5.6. Stability

The short-term stability of ampicillin in horse plasma was assessed in samples spiked at 2 µg/mL (in triplicates) and kept at 22 and 4 °C for 1, 3, 6, and 24 h. Long-term stability was evaluated in additional replicates stored at −20 °C for 4, 28, and 40 days and at −80 °C for 40 days. Mean concentration had to be within ±15% of the nominal value.

### 4.6. Method Application

To evaluate the suitability of the proposed method for monitoring target antibiotic concentrations, samples from patients hospitalized at our veterinary hospital were analyzed. Samples were collected 5 min before and 5 min after each administration during the first 48 h of treatment in different patient groups: 4 horses (1 female Thoroughbred, and 3 female Standardbred, with an age range of 6–20 years old and a weight range of 470–500 kg), 10 foals (4 females, 6 males, including 6 Standardbreds, 1 Mixbred, 1 Arabian, and 2 Quarter Horses, with an age range of 9 to 194.5 hours and a weight range of 26–66 kg) receiving 20 mg/kg of intravenous ampicillin every 8 and 6 h, respectively, and 2 dogs (1 male Australian Shepherd, 26 kg, 9 years old, and 1 male Italian Spinone, 42.5 kg, 12 years old) treated with a combination of ampicillin (20 mg/kg) and sulbactam (10 mg/kg) intravenously every 6 h. Blood collection from the jugular, saphenous, or cephalic veins for this study was approved by the Animal Welfare Committee of the University of Bologna, Prot. n. 358,467, ID n. 4626. Serum samples were obtained by centrifuging for 10 min at 3000× *g* and immediately transferred to plastic tubes. Plasma was separated through centrifugation for 30 min at 600× *g*. Both matrices were then stored at −80 °C until analysis.

## 5. Conclusions

In conclusion, the present study outlines a straightforward and efficient protocol that can be applied for the real-time therapeutic monitoring of ampicillin in dogs, horses, and foals, as well as other beta-lactam antibiotics, such as amoxicillin. The procedure begins with the critical step of timely sample collection, followed by transport to the laboratory, where accurate quantification is performed using LC-MS/MS. The correlation of timely quantified levels of antibiotics with clinical breakpoints or MIC data for the isolated pathogen, when available, would enable the swift implementation of therapeutic drug monitoring and optimization of dosing regimens for patients.

## Figures and Tables

**Table 1:** Intraday and interday accuracy and precision of ampicillin in horse plasma and dog serum.

	Horse		Dog	
	Accuracy (%)	Precision (%)	Accuracy (%)	Precision (%)
	LLOQ (0.1 µg/mL)		LLOQ (0.1 µg/mL)	
Day 1 (n = 5)	0.3	0.6	1.0	1.7
Day 2 (n = 5)	0.5	1.2	2.0	3.4
Day 3 (n = 5)	1.5	2.1	0.3	0.6
Interday (n = 15)	0.8	1.2	1.1	2.1
	QCL (0.5 µg/mL)		QCL (0.5 µg/mL)	
Day 1 (n = 5)	3.9	4.8	−0.7	1.2
Day 2 (n = 5)	9.3	12.9	0.0	2.0
Day 3 (n = 5)	−0.9	8.8	−1.3	2.3
Interday (n = 15)	4.1	9.3	−0.7	1.7
	QCM (5 µg/mL)		QCM (5 µg/mL)	
Day 1 (n = 5)	3.2	6.9	1.7	2.8
Day 2 (n = 5)	0.6	5.4	−1.7	2.9
Day 3 (n = 5)	0.2	7.3	0.0	8.7
Interday (n = 15)	1.3	5.9	0.0	5.0
	QCH (50 µg/mL)		QCH (50 µg/mL)	
Day 1 (n = 5)	−1.7	4.8	−6.2	5.3
Day 2 (n = 5)	2.7	2.3	−2.2	−10.5
Day 3 (n = 5)	4.5	1.3	8.8	3.1
Interday (n = 15)	1.9	3.8	0.2	9.0

**Table 2:** Intraday and interday accuracy and precision of sulbactam in dog serum.

	Dog	
	Accuracy (%)	Precision (%)
	QCL (0.5 µg/mL)	
Day 1 (n = 5)	0.3	0.6
Day 2 (n = 5)	1.0	1.7
Day 3 (n = 5)	0.7	1.1
Interday (n = 15)	0.7	1.1
	QCM (5 µg/mL)	
Day 1 (n = 5)	0.0	0.0
Day 2 (n = 5)	−5.0	0.0
Day 3 (n = 5)	−1.7	2.9
Interday (n = 15)	−2.2	2.7
	QCH (50 µg/mL)	
Day 1 (n = 5)	−3.3	3.0
Day 2 (n = 5)	−0.2	11.4
Day 3 (n = 5)	13.0	5.2
Interday (n = 15)	3.2	9.7

**Table 3:** Results of ampicillin, sulbactam, and amoxicillin-d4 ME, RE, and PE experiments, obtained from three replicates at three spike levels for each type of sample in horse plasma (A = standard calibrators in mobile phase, containing the same amount of analytes as the QC samples; B = blank samples of each matrix fortified with the same amount of analytes after extraction; C = samples of each matrix fortified with the same amount of analytes and extracted).

	Mean Peak Area (Arbitrary Units, ×10^3^, n = 3)			ME (%)	RE (%)	PE (%)
	A	B	C			
Ampicillin horse						
0.1 µg/mL	3.3	3.2	2.7	97.7	84.0	82.6
2 µg/mL	41.4	37.5	35.4	89.5	94.0	84.5
20 µg/mL	423.7	392.4	407.5	92.6	104.0	96.2
Ampicillin dog						
0.1 µg/mL	3.3	2.8	2.7	84.3	99.0	83.8
2 µg/mL	41.9	41.5	41.8	98.9	101.0	99.6
20 µg/mL	423.6	381.3	373.2	90.0	98.0	88.1
Sulbactam dog						
2 µg/mL	2.6	2.6	2.6	101.0	99.7	100.9
20 µg/mL	24.9	26.6	24.5	106.7	92.0	98.3

**Table 4:** Short-term and long-term stability of ampicillin in horse plasma, evaluated in samples spiked at 2 µg/mL (n = 3) across various temperatures and time points.

	1 h	3 h	6 h	24 h	4 d	28 d	40 d
22 °C	14.6%	−13.1%	−17.9	−40.2%	-	-	-
4 °C	8.3%	−3.3%	−8.2%	−10.3%	-	-	-
−20 °C	-	-	-	−7.7%	−3.2%	−12.8%	−9.8%
−80 °C	-	-	-	-	-	-	−5.3%

**Table 5:** Monitored transitions for each analyte, with correspondent cone voltage and collision energy values.

Analyte	MRM Transition (*m*/*z*)	Cone Voltage (V)	Collision Energy (eV)
Ampicillin	350.18 > 105.97	42	18
	350.18 > 113.97	42	30
Sulbactam	232.00 > 140.00	25	12
Piperacillin	518.35 > 143.04	40	18
	518.35 > 160.01	40	9
Tazobactam	299.10 > 138.00	25	13
Amoxicillin-d4	370.10 > 113.90	30	20
	370.10 > 212.00	30	12

## Data Availability

The original contributions presented in this study are included in the article. Further inquiries can be directed to the corresponding authors.

## References

[1] Cattaneo D., Gervasoni C., Corona A. (2022). The Issue of Pharmacokinetic-Driven Drug-Drug Interactions of Antibiotics: A Narrative Review. Antibiotics.

[2] Calvo M., Stefani S., Migliorisi G. (2024). Bacterial Infections in Intensive Care Units: Epidemiological and Microbiological Aspects. Antibiotics.

[3] Aslam B., Khurshid M., Arshad M.I., Muzammil S., Rasool M., Yasmeen N., Shah T., Chaudhry T.H., Rasool M.H., Shahid A. (2021). Antibiotic Resistance: One Health One World Outlook. Front. Cell. Infect. Microbiol..

[4] Caneschi A., Bardhi A., Barbarossa A., Zaghini A. (2023). The Use of Antibiotics and Antimicrobial Resistance in Veterinary Medicine, a Complex Phenomenon: A Narrative Review. Antibiotics.

[5] Lim J.M., Singh S.R., Duong M.C., Legido-Quigley H., Hsu L.Y., Tam C.C. (2020). Impact of National Interventions to Promote Responsible Antibiotic Use: A Systematic Review. J. Antimicrob. Chemother..

[6] Veringa A., Sturkenboom M.G.G., Dekkers B.G.J., Koster R.A., Roberts J.A., Peloquin C.A., Touw D.J., Alffenaar J.-W.C. (2016). LC-MS/MS for Therapeutic Drug Monitoring of Anti-Infective Drugs. TrAC Trends Anal. Chem..

[7] Kang J.-S., Lee M.-H. (2009). Overview of Therapeutic Drug Monitoring. Korean J. Intern. Med..

[8] Fang Z., Zhang H., Guo J., Guo J. (2024). Overview of Therapeutic Drug Monitoring and Clinical Practice. Talanta.

[9] Martin-Loeches I. (2022). Therapeutic Drug Monitoring (TDM) in Real-Time: A Need for the Present Future. Expert Rev. Anti-Infect. Ther..

[10] Voulgaridou G., Paraskeva T., Ragia G., Atzemian N., Portokallidou K., Kolios G., Arvanitidis K., Manolopoulos V.G. (2023). Therapeutic Drug Monitoring (TDM) Implementation in Public Hospitals in Greece in 2003 and 2021: A Comparative Analysis of TDM Evolution over the Years. Pharmaceutics.

[11] Junaid T., Wu X., Thanukrishnan H., Venkataramanan R., Thomas D. (2019). Chapter 30-Therapeutic Drug Monitoring. Clinical Pharmacy Education, Practice and Research.

[12] Ranjan G., Jamal F., Das S., Gupta V. (2023). Therapeutic Drug Monitoring: A Review. J. Drug Deliv. Ther..

[13] Gross A.S. (2001). Best Practice in Therapeutic Drug Monitoring. Br. J. Clin. Pharmacol..

[14] Märtson A.-G., Sturkenboom M.G.G., Stojanova J., Cattaneo D., Hope W., Marriott D., Patanwala A.E., Peloquin C.A., Wicha S.G., van der Werf T.S. (2020). How to Design a Study to Evaluate Therapeutic Drug Monitoring in Infectious Diseases?. Clin. Microbiol. Infect..

[15] EMA ICH M10 on Bioanalytical Method Validation-Scientific Guideline. https://www.ema.europa.eu/en/ich-m10-bioanalytical-method-validation-scientific-guideline.

[16] FDA Q2(R2) Guideline on Validation of Analytical Procedures Guidance for Industry. https://www.fda.gov/regulatory-information/search-fda-guidance-documents/q2r2-validation-analytical-procedures.

[17] Wilhelm A.J., den Burger J.C.G., Swart E.L. (2014). Therapeutic Drug Monitoring by Dried Blood Spot: Progress to Date and Future Directions. Clin. Pharmacokinet..

[18] Avataneo V., D’Avolio A., Cusato J., Cantù M., De Nicolò A. (2019). LC-MS Application for Therapeutic Drug Monitoring in Alternative Matrices. J. Pharm. Biomed. Anal..

[19] Shipkova M., Svinarov D. (2016). LC–MS/MS as a Tool for TDM Services: Where Are We?. Clin. Biochem..

[20] Seger C., Salzmann L. (2020). After Another Decade: LC-MS/MS Became Routine in Clinical Diagnostics. Clin. Biochem..

[21] Thomas S.N., French D., Jannetto P.J., Rappold B.A., Clarke W.A. (2022). Liquid Chromatography–Tandem Mass Spectrometry for Clinical Diagnostics. Nat. Rev. Methods Primers.

[22] Gaspar V.P., Ibrahim S., Zahedi R.P., Borchers C.H. (2021). Utility, Promise, and Limitations of Liquid Chromatography-Mass Spectrometry-Based Therapeutic Drug Monitoring in Precision Medicine. J. Mass Spectrom..

[23] Rahman M.M., Alam Tumpa M.A., Zehravi M., Sarker M.T., Yamin M., Islam M.R., Harun-Or-Rashid M., Ahmed M., Ramproshad S., Mondal B. (2022). An Overview of Antimicrobial Stewardship Optimization: The Use of Antibiotics in Humans and Animals to Prevent Resistance. Antibiotics.

[24] Kondampati K.D., Saini S.P.S., Sidhu P.K., Anand A., Kumar D., Beesam S., Bedi J.S., Kaur R., Bhardwaj R. (2022). Pharmacokinetic-Pharmacodynamic Study of Ampicillin-Cloxacillin Combination in Indian Thoroughbred Horses (Equus Caballus) and Safety Evaluation of the Computed Dosage Regimen. J. Equine Vet. Sci..

[25] Monaghan K., Labato M., Papich M. (2021). Ampicillin Pharmacokinetics in Azotemic and Healthy Dogs. J. Vet. Intern. Med..

[26] Bardhi A., Gazzotti T., Pagliuca G., Mari G., Barbarossa A. (2022). Validation of a Single Liquid Chromatography-tandem Mass Spectrometry Approach for Oxytetracycline Determination in Bull Plasma, Seminal Plasma and Urine. Drug Test. Anal..

[27] Barbarossa A., Bardhi A., Gazzotti T., Mari G., Pagliuca G. (2022). A Single LC-MS/MS Validated Method for Tulathromycin Quantification in Plasma, Seminal Plasma, and Urine to Be Applied in a Pharmacokinetic Study in Bull. Drug Test. Anal..

[28] Bardhi A., Romano J.E., Pagliuca G., Caneschi A., Barbarossa A. (2023). Florfenicol and Florfenicol Amine Quantification in Bull Serum and Seminal Plasma by a Single Validated UHPLC-MS/MS Method. Vet. Med. Int..

[29] Van den Hoven R., Hierweck B., Dobretsberger M., Ensink J.M., Meijer L.A. (2003). Intramuscular Dosing Strategy for Ampicillin Sodium in Horses, Based on Its Distribution into Tissue Chambers before and after Induction of Inflammation. J. Vet. Pharmacol. Ther..

[30] Robbins S.N., Goggs R., Lhermie G., Lalonde-Paul D.F., Menard J. (2020). Antimicrobial Prescribing Practices in Small Animal Emergency and Critical Care. Front. Vet. Sci..

[31] Escher M., Vanni M., Intorre L., Caprioli A., Tognetti R., Scavia G. (2011). Use of Antimicrobials in Companion Animal Practice: A Retrospective Study in a Veterinary Teaching Hospital in Italy. J. Antimicrob. Chemother..

[32] Black D.M., Rankin S.C., King L.G. (2009). Antimicrobial Therapy and Aerobic Bacteriologic Culture Patterns in Canine Intensive Care Unit Patients: 74 Dogs (January–June 2006). J. Vet. Emerg. Crit. Care.

[33] Buckland E.L., O’Neill D., Summers J., Mateus A., Church D., Redmond L., Brodbelt D. (2016). Characterisation of Antimicrobial Usage in Cats and Dogs Attending UK Primary Care Companion Animal Veterinary Practices. Vet. Rec..

[34] Schmitt K., Lehner C., Schuller S., Schüpbach-Regula G., Mevissen M., Peter R., Müntener C.R., Naegeli H., Willi B. (2019). Antimicrobial Use for Selected Diseases in Cats in Switzerland. BMC Vet. Res..

[35] Chinemerem Nwobodo D., Ugwu M.C., Oliseloke Anie C., Al-Ouqaili M.T.S., Chinedu Ikem J., Victor Chigozie U., Saki M. (2022). Antibiotic Resistance: The Challenges and Some Emerging Strategies for Tackling a Global Menace. J. Clin. Lab. Anal..

[36] Odenholt I., Gustafsson I., Löwdin E., Cars O. (2003). Suboptimal Antibiotic Dosage as a Risk Factor for Selection of Penicillin-Resistant Streptococcus Pneumoniae: In Vitro Kinetic Model. Antimicrob. Agents Chemother..

[37] Póvoa P., Moniz P., Pereira J.G., Coelho L. (2021). Optimizing Antimicrobial Drug Dosing in Critically Ill Patients. Microorganisms.

[38] Mabilat C., Gros M.F., Nicolau D., Mouton J.W., Textoris J., Roberts J.A., Cotta M.O., van Belkum A., Caniaux I. (2020). Diagnostic and Medical Needs for Therapeutic Drug Monitoring of Antibiotics. Eur. J. Clin. Microbiol. Infect. Dis..

[39] Nair A.B., Jacob S. (2016). A Simple Practice Guide for Dose Conversion between Animals and Human. J. Basic Clin. Pharm..

[40] Nahata M.C., Vashi V.I., Swanson R.N., Messig M.A., Chung M. (1999). Pharmacokinetics of Ampicillin and Sulbactam in Pediatric Patients. Antimicrob. Agents Chemother..

[41] Lee H.J., Ryu P.D., Lee H., Cho M.H., Lee M.H. (2001). Screening for Penicillin Plasma Residues in Cattle by Enzyme-Linked Immunosorbent Assay. Acta Vet. Brno.

[42] McWhinney B.C., Wallis S.C., Hillister T., Roberts J.A., Lipman J., Ungerer J.P.J. (2010). Analysis of 12 Beta-Lactam Antibiotics in Human Plasma by HPLC with Ultraviolet Detection. J. Chromatogr. B-Anal. Technol. Biomed. Life Sci..

[43] Carlier M., Stove V., De Waele J.J., Verstraete A.G. (2015). Ultrafast Quantification of β-Lactam Antibiotics in Human Plasma Using UPLC-MS/MS. J. Chromatogr. B-Anal. Technol. Biomed. Life Sci..

[44] Sturgeon C.M., Viljoen A. (2011). Analytical Error and Interference in Immunoassay: Minimizing Risk. Ann. Clin. Biochem..

[45] Schreiber G., Keating A.E. (2011). Protein Binding Specificity versus Promiscuity. Curr. Opin. Struct. Biol..

[46] Strathmann F.G., Hoofnagle A.N. (2011). Current and Future Applications of Mass Spectrometry to the Clinical Laboratory. Am. J. Clin. Pathol..

[47] Tang L., Swezey R.R., Green C.E., Mirsalis J.C. (2022). Enhancement of Sensitivity and Quantification Quality in the LC-MS/MS Measurement of Large Biomolecules with Sum of MRM (SMRM). Anal. Bioanal. Chem..

[48] Rankin-Turner S., Heaney L.M. (2023). Mass Spectrometry in the Clinical Laboratory. A Short Journey through the Contribution to the Scientific Literature by CCLM. Clin. Chem. Lab. Med. (CCLM).

[49] Bardhi A., Zaghini A., Levionnois O., Barbarossa A. (2021). A Quick Approach for Medetomidine Enantiomer Determination in Dog Plasma by Chiral Liquid Chromatography-Tandem Mass Spectrometry and Application to a Pharmacokinetic Study. Drug Test. Anal..

[50] Romano J.E., Bardhi A., Pagliuca G., Villadόniga G.B., Barbarossa A. (2024). Pharmacokinetics of Florfenicol in Serum and Seminal Plasma in Beef Bulls. Theriogenology.

[51] Bardhi A., Vecchiato C.G., Sabetti M.C., Tardo A.M., Vasylyeva K., Biagi G., Pietra M., Barbarossa A. (2024). A Novel UHPLC–MS/MS Method for the Measurement of 25-Hydroxyvitamin D3 in Canine Serum and Its Application to Healthy Dogs. Animals.

[52] Schmerold I., van Geijlswijk I., Gehring R. (2023). European Regulations on the Use of Antibiotics in Veterinary Medicine. Eur. J. Pharm. Sci..

[53] Zakaria R., Allen K.J., Koplin J.J., Roche P., Greaves R.F. (2016). Advantages and Challenges of Dried Blood Spot Analysis by Mass Spectrometry across the Total Testing Process. J. Int. Fed. Clin. Chem. Lab. Med..

[54] Samsonova J.V., Saushkin N.Y., Osipov A.P. (2022). Dried Blood Spots Technology for Veterinary Applications and Biological Investigations: Technical Aspects, Retrospective Analysis, Ongoing Status and Future Perspectives. Vet. Res. Commun..

[55] Allaway D., Alexander J.E., Carvell-Miller L.J., Reynolds R.M., Winder C.L., Weber R.J.M., Lloyd G.R., Southam A.D., Dunn W.B. (2022). Suitability of Dried Blood Spots for Accelerating Veterinary Biobank Collections and Identifying Metabolomics Biomarkers With Minimal Resources. Front. Vet. Sci..

[56] Wickremsinhe E.R., Perkins E.J. (2015). Using Dried Blood Spot Sampling to Improve Data Quality and Reduce Animal Use in Mouse Pharmacokinetic Studies. J. Am. Assoc. Lab. Anim. Sci..

[57] Dunkel B., Johns I.C. (2015). Antimicrobial Use in Critically Ill Horses. J. Vet. Emerg. Crit. Care.

[58] (2019). EMA/CVMP/CHMP/682198/2017. Categorisation of Antibiotics for Use in Animals: Answer to the Request from the European Commission for Updating the Scientific Advice on the Impact on Public Health and Animal Health of the Use of Antibiotics in Animals. https://www.ema.europa.eu/en/documents/report/categorisation-antibiotics-european-union-answer-request-european-commission-updating-scientific-advice-impact-public-health-and-animal-health-use-antibiotics-animals_en.pdf.

[59] Taylor S. (2015). A Review of Equine Sepsis. Equine Vet. Educ..

[60] Theelen M.J., David Wilson W., Dacvim H., Byrne B.A., Edman J.M., Gary Magdesian K. (2016). Cumulative Antimicrobial Susceptibility of Bacteria Isolated From Foals With Sepsis: 1990–2015.

[61] Matuszewski B.K. (2006). Standard Line Slopes as a Measure of a Relative Matrix Effect in Quantitative HPLC–MS Bioanalysis. J. Chromatogr. B.

